# Implementation of population screening for colorectal cancer by repeated Fecal Immunochemical Test (FIT): third round

**DOI:** 10.1186/1471-230X-12-73

**Published:** 2012-06-19

**Authors:** Inge Stegeman, Thomas R de Wijkerslooth, Rosalie C Mallant-Hent, Karin de Groot, An K Stroobants, Paul Fockens, Marco Mundt, Patrick MM Bossuyt, Evelien Dekker

**Affiliations:** 1 Department of Clinical Epidemiology, Biostatistics and Bioinformatics, Academic Medical Center, Amsterdam, The Netherlands; 2 Department of Gastroenterology and Hepatology, Academic Medical Center, Amsterdam, The Netherlands; 3 Department of Gastroenterology and Hepatology, Flevohospital, Almere, The Netherlands; 4 Department of Clinical Chemistry, Academic Medical Centre, Amsterdam, The Netherlands

## Abstract

**Background:**

Colorectal cancer (CRC) is the most common cancer in Europe with a mortality rate of almost 50%. The prognosis of patients is largely determined by the clinical and pathological stage at the time of diagnosis. Population screening has been shown to reduce CRC-related mortality rate. Most screening programs worldwide rely on fecal immunochemical testing (FIT). The effectiveness of a FIT screening program is not only influenced by initial participation rate, but also by program adherence during consecutive screening rounds. We aim to evaluate the participation rate in and yield of a third CRC screening round using FIT.

**Methods and design:**

Four years after the first screening round and two years after the second round, a total number of approximately 11,000 average risk individuals (50 to 75 years of age) will be invited to participate in a third round of FIT-based CRC screening. We will select individuals in the same target area as in the previous screening rounds, using the electronic database of the regional municipal administration registrations. We will invite all FIT-negatives and all non-participants in previous screening rounds, as well as eligible first time invitees who have moved into the area or have become 50 years of age.

FITs will be analyzed in the special technique laboratory of the Academic Medical Center of the University of Amsterdam. All FIT-positives will be invited for a consultation at the outpatient clinic. In the absence of contra-indications, a colonoscopy will follow at the Academic Medical Center or at the Flevohospital. The primary outcome measures are the participation rate, defined as the proportion of invitees that return a FIT in this third round of FIT-screening, and the diagnostic yield of the program.

**Implications:**

This study will provide precise data on the participation in later FIT screening rounds. This enables to estimate the effectiveness of CRC screening programs that rely on repeated FIT- screening, such as the one that will be implemented in the Netherlands in 2013.

## Background

Colorectal cancer (CRC) is the most common cancer in Europe. In 2008, there were an estimated 436,000 new cases of CRC and 212,000 deaths [1]. Currently, about five percent of persons at average risk develop CRC [2]. The prognosis of patients with CRC is largely influenced by the cancer stage at diagnosis [3]. Timely detection of cancer and removal of its precursor lesions (i.e. adenomas) has been shown to result in a lower CRC incidence and mortality [4,5]. Based on these arguments, screening for CRC meets the Wilson and Jungner criteria for population screening [6].

Several methods are available for population screening for CRC, which can be broadly itemized into stool tests and structural exams [7]. Stool tests include the guaiac, the immunochemical and DNA-marker tests. Structural exams comprise colonoscopy, sigmoidoscopy and CT colonography. The fecal occult blood test (FOBT) and sigmoidoscopy are the only screening methods with a documented mortality reduction over a follow-up period of 10 years. A meta-analysis of FOBT-based screening trials showed a 16% colorectal cancer mortality reduction in FOBT screening after several screening rounds [8]. A large randomized controlled trial in which subjects received either once-only sigmoidoscopy screening or no screening reported a 31% CRC-related mortality reduction in the screening group [9].

The Council of the European Union has recommended population screening for CRC by FOBT [10]. Several EU member states have adopted this advice, although some have implemented non-population based screening programs using other strategies, such as Poland and Germany. In the Netherlands, the effectiveness of FOBT screening has been intensively studied in the past 5 years. Two large randomized controlled trials were performed in the Amsterdam, Nijmegen en Rotterdam region, comparing participation rates of invitational population-based screening using either guiac-FOBT or immunochemical FOBT (FIT) [11,12]. Both trials demonstrated significantly higher participation rates in FIT than in guiac-FOBT based screening: 60% and 62% versus 47% and 50%. In addition, detection rates of advanced neoplasia (CRC and advanced adenomas) were higher in FIT screening than in guiac-based screening: 2.4% and 5.5% versus 1.1% and 1.2%. The effectiveness of population screening is largely determined by the participation rate, not only on participation in the initial screening round, but also on participation in consecutive rounds (“program adherence”). Two years after the first screening round in Amsterdam, 10,265 individuals received an invitation to participate in a second round FIT- screening [13]. Compared to the first round, a significant lower overall participation rate was observed in the second round (43% vs 52%). Overall, 86% of first round participants participated again in the second round and of the previous non-participants, 21% did participate in the second round [14].

Although second round participation in this program was lower, a further decrease is not necessary. In three consecutive screening rounds by guiac-based FOBT screening in Scotland, for example, participation remained stable: 55% participated in a first round, 53% in a second round, and 55% in a third round [15]. Two years after the second round and four years after the first round of FIT screening, members of the same dynamic Amsterdam cohort will once again receive an invitation to participate in population screening by FIT. We will document the level of participation and the yield of screening in this third round of FIT-based screening.

## Methods and design

### Objectives

#### Primary objective

To evaluate the participation rate in and yield of a third round of FIT-based colorectal cancer screening in the Netherlands, two years after the second round and four years after the first round.

### Secondary objectives

We will compare overall participation rates in the third round with those in the first and second round and will compare participation rates in previous participants and non-participants of the first and/or second round. In addition, we will evaluate the complication rate of FIT screening, including follow-up colonoscopy in FIT-positives, and compare these to the results of the first and second round of screening. Also we will evaluate the number and cancer stage of interval cancers in the cohort during the 3 consecutive rounds.

### Study design and population

This study is a designed as a dynamic cohort study. The cohort consists of approximately 11,000 men and women between 50 and 75 years of age at average risk for CRC, living in the Amsterdam region, the same area as in the first and the second round. We will identify cohort members using the electronic database of the regional municipal administration registration (Gemeentelijke Basis Administratie (GBA)).

Institutionalized people will be excluded from participation. Participants in the first or second round who have previously tested positive will not be invited. We will instruct all invitees with rectal blood loss and or change in bowel habits not to participate in screening.

### Invitation procedure

Eligible cohort members will be invited between July 2011 and December 2011. All invitations will be sent out by the regional Comprehensive Cancer Centre Amsterdam using the same centralized invitation program as in previous screening rounds (ICOLON). This institution is also involved in the existing population-based screening programs in the region including breast and cervical cancer screening. Invitees will receive a pre-announcement, followed by an invitation kit 2 weeks later by postal mail containing an invitation letter, an information leaflet, an immunochemical FOBT, a test instruction and a frequently asked questions (FAQ) card. A reminder will be sent two and eight weeks, after the initial invitation.

### Information brochure

The information brochure covers information relevant to colorectal cancer screening. It contains facts on CRC in general, instructions how to perform the FIT, information regarding false-negative and false-positive results, details of the follow-up colonoscopy for FIT-positives, and information on the benefits and harms of screening. This leaflet is an updated version of the leaflet used in the second round. The leaflet is based on the principles of informed-choice, aiming designed to enable all invited persons to make a well-informed decision whether or not to participate.

### Informed consent

An informed consent form is sent together with the FIT. Before analyzing the stool sample, we will check if the informed consent is enclosed and signed. If the informed consent is not correctly available, the stool sample will not be analyzed and the participant will be notified by mail and asked to complete the informed consent.

### FIT

For this study the same FIT as the one used in previous rounds will be used (OC-sensor; Eiken Chimical Co, Tokyo, Japan). This test provides a quantitative measurement of human hemoglobin in stool. The FIT consist of a probe attached to a cap, which fits a collection tube containing hemoglobin stabilizing buffer. Participants are instructed to collect one bowel movement and to sweep the tip of the probe several times through the feces and to insert the probe into the collection tube afterwards. No diet restrictions are advised. In the specialized laboratory of the Academic Medical Center, the returned FITs will be processed using the OC- Sensor - automated instrument. According to previous rounds a subject is considered positive at a cut-off level of 50 ng hemoglobin per milliliter feces.

## Test results

The test result will be sent to the participant and his or her general practitioner by regular mail. In case of a positive test result, the participant will be invited for a consultation at the screening center at the Academic Medical Center in Amsterdam or the Flevo Hospital in Almere. During the consultation, the need for a follow-up colonoscopy will be discussed and the individuals exclusion criteria and/or contra-indications for colonoscopy are checked. Test positive Invitees with end-stage disease and a life-expectancy of less than 5 years, and those who have had a complete colonoscopy in the previous two years, will not be invited for colonosopy. In absence of contra-indications, a colonoscopy will be planned within two weeks after the consultation. In case of a negative test result, no follow up is advised.

### Colonoscopy

Colonoscopies are performed at the Academic Medical Center or at the Flevohospital. All colonoscopies will be performed by experienced endoscopists (≥ 200 colonoscopies) according to the prevailing quality guidelines [16]. Colonoscopy quality indicators will be recorded on a case record form. In case of polyps or cancer, endoscopic removal of the lesions will be attempted during the same procedure. If endoscopic removal is not possible, biopsies will be obtained and histopathological assessment will provide a definitive diagnosis.

### Lesions and pathology

Data on location, size, macroscopic aspect, morphology type of procedure (diagnostic or therapeutic) and endoscopic assessment of radicality will be recorded for all lesions detected during colonoscopy. Lesions will be evaluated by an experienced gastrointestinal pathologist according to the Vienna criteria [17]. All lesions will be classified as adenoma (tubular, tubulovillous, villous), serrated (hyperplastic, sessile serrated adenoma, traditional serrated adenoma), adenocarcinoma or miscellaneous. Dysplasia will be defined as either low-grade or high-grade.

### Follow-up after colonoscopy

Colonoscopy findings will be discussed with the participant by telephone or at the outpatient clinic, if desired, two weeks after the colonoscopy. If the colonoscopy is negative, no follow up is advised. In case of polyps, surveillance colonoscopy is advised according to the Dutch surveillance guidelines [18]. In case of CRC, the patient will be invited at the outpatient clinic and referred to a gastroenterologist or surgeon for further treatment. The general practitioner will be informed about the colonoscopy result by mail.

### Cancer registry

All invitees are linked to The Netherlands Cancer Registry, which collects data of new cancer patients, such as tumor type, incidence date and stage [19]. Using this registry, we can identify interval carcinomas in the three consecutive screening rounds.

### Ethical approval

Ethical approval was obtained from the Dutch Health Council (The Hague, The Netherlands).

### Data analysis

Participation rate is defined as the number of invitees returning a FIT relative to the number of all eligible invitees. The third round participation rate will be compared with those of the two previous screening rounds. Participation rates in subgroups of participants and non-participants of previous screening rounds will be compared. Among third round invitees the following subgroups can be defined: (1) non-participants in the first and the second round; (2) participants in the first and the second round; (3) invitees who participated in the first but did not in the second round; (4) second round participants who did not participate in the first round (5) second round participants who were not invited for the first round (6) second round non-participants who were not invited for the first round and (7) first time invitees. Screening yield is calculated by dividing the total number of participants with detected advanced neoplasia by the number of participants (per-protocol analysis) and the total number of invitees (intention-to-screen analysis). Advanced neoplasia is defined as a CRC or an advanced adenoma. An advanced adenoma is defined as an adenoma ≥ 10 mm, an adenoma with ≥ 25% villous histology or an adenoma with high grade dysplasia. We will express participation rates and diagnostic yield as proportions and present estimates with corresponding confidence intervals. We will compare proportions by Chi-square statistic. We will evaluate the number and stage of interval cancers. Interval cancers will be defined as the proportion of cancer diagnosed in first and second round participants outside the screening. Interval cancers will be identified via cross-linkage of the screening pilot with the Dutch cancer registry. Cancer stage at diagnosis and location will be retrieved via the registry. Descriptive statistics will be used to analyze the interval cancers. Chi-square test statistic will be used to analyze the distribution of cancer stage and location between screen detected cancers of the first, second and third round and interval cancers.

### Implications

This study will provide information on participation and yield in the third round of a FIT- based CRC screening program. Participation rate and determinants of participants will be compared with the first and the second screening round. This study will also provide information on interval cancers in FIT based screening, in a third round of biannual pilot screening. As FIT is generally preferred over guiaic-FOBT in most countries FIT will become the test of choice. The study results will enable us to estimate the effectiveness of a FIT-based CRC screening program with 2-yearly rounds, such as the one that will be implemented in the Netherlands in 2013.

## Abbreviations

CRC, Colorectal cancer; FOBT, Fecal occult blood test; EU, European union; FIT, Fecal immunochemical test.

## Competing interests

The authors declare that they do not have competing interests.

## Authors’ contributions

IS and TW are responsible for the drafting of this manuscript. IS and KG are responsible for the logistics of the screening program. ED, PB, RM and MM are responsible for the study design and the revision of the manuscript. All authors have read and approved the manuscript.

## Pre-publication history

The pre-publication history for this paper can be accessed here:

http://www.biomedcentral.com/1471-230X/12/73/prepub
